# An algebraic approach to circulant column parity mixers

**DOI:** 10.1007/s10623-024-01476-w

**Published:** 2024-08-21

**Authors:** Robert Christian Subroto

**Affiliations:** Digital Security, Radboud University, Nijmegen, The Netherlands

**Keywords:** Column parity mixers, Module theory, Local rings, Linear algebra, Circulant matrices, 13P25, 13M05, 13M10, 68R01

## Abstract

Circulant Column Parity Mixers (CCPMs) are a particular type of linear maps, used as the mixing layer in permutation-based cryptographic primitives like Keccak-*f* (SHA3) and Xoodoo. Although being successfully applied, not much is known regarding their algebraic properties. They are limited to invertibility of CCPMs, and that the set of invertible CCPMs forms a group. A possible explanation is due to the complexity of describing CCPMs in terms of linear algebra. In this paper, we introduce a new approach to studying CCPMs using module theory from commutative algebra. We show that many interesting algebraic properties can be deduced using this approach, and that known results regarding CCPMs resurface as trivial consequences of module theoretic concepts. We also show how this approach can be used to study the linear layer of Xoodoo, and other linear maps with a similar structure which we call DCD-compositions. Using this approach, we prove that every DCD-composition where the underlying vector space with the same dimension as that of Xoodoo has a low order. This provides a solid mathematical explanation for the low order of the linear layer of Xoodoo, which equals 32. We design a DCD-composition using this module-theoretic approach, but with a higher order using a different dimension.

## Introduction

Column parity mixers [9], or CPMs for short, are a particular type of linear maps which are a generalization of the $\theta$ mixing layers in the cryptographic permutations Xoodoo [4] and Keccak-*f* [3]. They provide a good trade-off between implementation cost and mixing power, making them well suited for lightweight cryptography.

A formal approach in studying CPMs as a stand alone topic is done in [9], where CPMs were formulated as linear maps between spaces of matrices. Each CPM $\theta$, viewed as an endomorphism of the ring of $m \times n$-matrices, is uniquely determined by an $n \times n$-matrix called the parity folding matrix of $\theta$. There has been some emphasis on studying CPMs where its parity folding matrix belongs to the class of circulant matrices (see [5] for more details about circulant matrices). These CPMs are called circulant CPMs which we abbreviate by CCPMs. Due to the symmetric properties of circulant matrices, CCPMs have a good worst-case behaviour for the purpose of mixing bits. The $\theta$ mixing layers of Xoodoo and Keccak-*f* are examples of CCPMs.

In [9], a criterion was provided to determine the invertibility of a CCPM by studying the corresponding parity folding matrix. It was also shown that the set of invertible CCPMs forms a group. This is everything that is known so far about the algebraic properties of CCPMs. A reason might be the complexity of describing CCPMs in terms of linear algebra, which at first glance might indicate that no strong conclusions can be drawn regarding their algebraic structure. It turns out that viewing CCPMs as *R*-module homomorphisms, where *R* is the ring of circulant matrices, is very effective in studying CCPMs. As a result, many interesting properties can be extracted with this approach, and some known results like the invertibility criterion resurfaced as trivial concepts from module theory.

The order of the linear layer in the round function of a cryptographic primitive is relevant in the resistance against invariant subspace attacks, where a low order indicates a potential weakness [2]. The linear layer of Xoodoo is a composition of a circulant bit permutation, a CCPM and another circulant bit permutation, and it was numerically determined that the linear layer has an order of only 32. A mathematical explanation for this low order however remained absent. As it turns out, such an explanation can be found by using the module theoretic approach which we used for studying CCPMs. It would be interesting to know if we can find variants of the linear layer of Xoodoo with a higher order, by means of finding new compositions, and/or by changing the dimensions of the state of the permutations.

### Outline

In Sect. 2, we present a mathematical framework based on commutative algebra as a foundation to studying column parity mixers, which includes module theory and localization of rings.

In Sect. 3, we introduce circulant rings, which are a generalization of the ring of circulant matrices. We provide a full classification of local circulant rings, as well the corresponding algebraic properties. Moreover, we give a geometric interpretation of circulant rings by considering free modules over these type of rings.

In Sect. 4, we introduce a generalization of CCPMs where we define them as *R*-linear maps of free *R*-modules, where *R* is a commutative ring with unity. We exploit the algebraic properties of *R* to gain a deeper understanding of the algebraic structure of CCPMs. These include a full description of the eigenspaces of a CCPM viewed as an *R*-module homomorphism, and the order of a CCPM.

In Sect. 5, we show that the linear layer of Xoodoo can be interpreted as an $R_{4,32}$-linear map of the free 3-dimensional $R_{4,32}$-module $R_{4,32}^3$, where $R_{4,32}$ is a local circulant ring. We introduce DCD-compositions, which are compositions with a similar structure to that of the linear layer of Xoodoo. We use the results of Sects. 3 and 4 to construct DCD-compositions with a higher order.

In Sect. 6, we briefly discuss some potential cryptographic implications of the new module theoretic setting discussed in Sect. 3.

#### Contributions

The main contributions of this paper are the results presented in Sects. 3, 4 and 5. We introduced circulant rings, and we provided a natural construction of modules over circulant rings, which we call circulant modules. A major new result in this paper is that CCPMs are in fact module homomorphisms over free circulant modules, which provides deeper insight in the algebraic structure of CCPMs. We showed using this observation that some known algebraic properties of CCPMs become trivial consequences. Think of for example the invertibility criterion, and the group properties of invertible CCPMs. Moreover, we also show how this new approach gives us deeper insight in the eigenvectors and eigenvalues of CCPMs, which has not been done before.

Another contribution of this paper is that we provide a solid mathematical explanation for the low order of the linear layer of Xoodoo. We achieved this by showing that not only the $\theta$ map, but the whole linear layer is in fact a module homomorphism of circulant modules. By doing so, we demonstrate how the order relates to the ring structure of the underlying circulant ring. Moreover, we show how this approach allows us to design linear mappings with a similar design philosophy of the linear layer of Xoodoo, but where its order is significantly higher. Its cryptographic relevance lies in the security against invariant subspace attacks [2].

#### Notation

The **cardinality** of a set *S* is denoted as $\# S$. The set of all positive integers strictly greater than 0 is denoted as $\mathbb {Z}_{>0}$.

Given a commutative ring *R* with unity, we denote the **multiplicative group of invertible elements** of *R* by $R^*$. We refer to ${\text {Spec}}(R)$ as the **set of all proper prime ideals**, and ${\text {MaxSpec}}(R)$ as the **set of all maximal ideals** of *R*. Given an ideal $\mathfrak {a}$ in *R*, the **radical** of $\mathfrak {a}$ is denoted by $r(\mathfrak {a})$.

The **ring- or set of all** $m \times m$-**matrices over ring** *R* is denoted by ${\text {M}}_m(R)$. The **multiplicative group of** $m \times m$-**invertible matrices over a ring** *R* is denoted by ${\text {GL}}_m(R)$. Moreover, ${\text {SL}}_m(R)$ is the set of matrices $M \in {\text {GL}}_m(R)$ where $\det (A) = 1_R$. For a matrix $A \in {\text {M}}_m(R)$, we say that $A_{ij}$ is the **entry in the** *i*-**th row and** *j*-**th column**. Here we use the convention that the **indexing of coordinates** runs from 0 to $m-1,$ hence $0 \le i,j \le m-1$. We refer to $I_m$ as the **identity matrix**, and $0_{m \times m}$ as the **zero matrix** in ${\text {M}}_m(R)$.

For $\mathbb {F}$ a field, we denote $\mathbb {F}^n$ as the *n*-dimensional vector space over $\mathbb {F}$. Its vectors are considered as **column (vertical) vectors**, unless stated otherwise. We **index the coordinates** of a (column) vector $v \in \mathbb {F}^n$ from 0 to $n-1$. Naturally, for $0 \le i \le n-1$, $v_i$ is the *i*-th coordinate of *v*. We denote the **transpose** of *v* by $v^{\textsf{T}}$. If for example *v* is a row vector, $v^{\textsf{T}}$ is a column vector. We refer to $\textsf{e}_y$ as the *y*-**th standard unit vector of** $\mathbb {F}^n$ where $0 \le y \le n-1$. The zero vector is defined as $0_n$.

We denote ${\text {Tor}}(G)$ as the **torsion subgroup** of a group *G*, i.e. the elements in *G* with finite order. For *f* in some finite group *G*, we denote the **order** of *f* by ${\text {ord}}(f)$. When $G = (\mathbb {Z}/ m \mathbb {Z})^*$, we denote the **multiplicative order** of $g \in (\mathbb {Z}/ m \mathbb {Z})^*$ as ${\text {ord}}_m(g)$. Moreover, $\gcd$ and ${\text {lcm}}$ represent the **greatest common divisor** and the **least common multiple** respectively.

## Algebraic framework

This section contains a brief summary of the required algebraic prerequisites for our research. We heavily rely on concepts from commutative algebra. See [1, 6, 8] for a more detailed treatment of these topics.

### Local rings and localization

An important class of rings in commutative algebra are local rings. A ring *R* is called **local** if it has a unique maximal ideal, which we denote by $\mathfrak {m}$. The field $\mathbb {F}:= R / \mathfrak {m}$ is defined as the **residue field** of *R*. We have the natural quotient map

1 $$\begin{aligned} q_R :R \rightarrow R / \mathfrak {m}\cong \mathbb {F}, \ r \mapsto r \bmod \mathfrak {m}. \end{aligned}$$

To ease notation, we denote $q_R(r) = r \bmod \mathfrak {m}$ as $\overline{r}$. For local rings, it is known that $r \in R$ is invertible if and only if $r \notin \mathfrak {m}$.

Local rings have been well studied in commutative algebra, resulting into many interesting properties. For example, finitely generated modules over a local ring are closely related to vector spaces over fields due to Nakayama’s Lemma [1]. We extend these properties to non-local commutative rings by applying a technique called **localization**, which is a technique where given a non-local ring *R* and for a chosen $\mathfrak {p}\in {\text {Spec}}(R)$, one can construct a local ring $R_{\mathfrak {p}}$ where its maximal ideal is denoted by $\mathfrak {p}\cdot R_{\mathfrak {p}}$. Intuitively, we make all elements in *R* outside $\mathfrak {p}$ invertible. Details about this construction can be found in [1]. For now, it suffices to know that we have a natural ring homomorphism

$$\begin{aligned} l_{\mathfrak {p}} :R \rightarrow R_{\mathfrak {p}}, \ r \mapsto \frac{r}{1_R}. \end{aligned}$$

More about local rings and localization can be found in many books treating commutative algebra, like [1, 6].

### Modules and linear algebra

Modules can be considered as a generalization of vector spaces. In its most general form, it is defined as follows:

#### Definition 1

[1] Let *R* be a ring. An *R*-**module** consists of the pair $(V,\mu )$ where *V* is a commutative group and $\mu$ is a mapping of $R \times V$ to *V* such that, if we write *ax* for $\mu (a,x)$ where $a \in R$ and $x \in V$, the following properties are satisfied:

$$\begin{aligned} r(x+y)&= rx + ry \\ (r_1 + r_2)x&= r_1 x + r_2 x \\ (r_1 r_2)x&= r_1( r_2 x) \\ 1x&= x \ \ \ \ \ \ \ \ \ \ \ \ \ \ \ \ \ \ \ \ (r, r_1, r_2 \in R \text { and } x,y \in V). \end{aligned}$$

#### Remark 1

A trivial but important example of an *R*-module is the ring *R* itself, considered as a commutative group under addition, and where $\mu :R \times R \rightarrow R$ is the multiplication map.

#### Definition 2

[1] An *R*-**submodule** $V'$ of *V* is a subgroup of *V* such that $r \cdot v' \in V'$ for all $r \in R$ and $v' \in V'$.

#### Example 1

Let $\mathfrak {a}$ be an ideal of *R* and define

$$\begin{aligned} \mathfrak {a}V := \left\{ \sum _{i=0}^t a_i \cdot v_i \mid a_i \in \mathfrak {a}, v_i \in V, t \in \mathbb {Z}_{>0} \right\} , \end{aligned}$$

which in words means that $\mathfrak {a}V$ consists of finite sums of terms of the form $a \cdot v$ where $a \in \mathfrak {a}$ and $v \in V$. Unless $V = \{ 0 \}$, we have that $\mathfrak {a}V$ is in many cases a proper *R*-submodule of *V*. Nakayama’s Lemma [1] is very useful in studying these types of submodules.

We are mainly interested in **free modules** of finite rank. An *R*-module *V* if called **free** of rank *m* if there exist elements $\textsf{e}_0, \dotsc ,\textsf{e}_{m-1} \in V$ such that every element $v \in V$ is **uniquely** expressed as

2 $$\begin{aligned} v = \sum _{i=0}^{m-1} r_i \cdot \textsf{e}_i \ \ \ \ \ \ \ \ \ \ r_i \in R. \end{aligned}$$

In algebraic terms, a free module *V* of rank *m* is of the form $V = \bigoplus _{i=0}^{m-1} R$, which we also denote as $R^{m}$. We call $\{ \textsf{e}_0, \dotsc ,\textsf{e}_{m-1} \}$ an *R*-**basis** of *V*. By fixing a basis, every element $v \in V$ is represented by the column vector $v = (r_0, \dotsc ,r_{m-1})^{\textsf{T}}$, where addition is defined coordinate-wise.

### Endomorphisms

Free modules have a lot in common with vector spaces. Not only because of the unique representation of elements as in (2), but also in terms of linear transformations.

#### Definition 3

[1] Let $V_1$ and $V_2$ be *R*-modules. Then an *R*-**linear map** from $V_1$ to $V_2$ is a map $\theta :V_1 \rightarrow V_2$ such that for all $a,b \in V_1$ and $r \in R$, we have

$$\begin{aligned} \theta (a + b)&= \theta (a) + \theta (b) \\ \theta (ra)&= r \theta (a). \end{aligned}$$

When $\theta$ is bijective, we say that $\theta$ is an *R*-**isomorphism**. In the special case when $V_1 = V_2 = V$, we say that $\theta$ is an *R*-**endomorphism** of *V*, and we denote the set of all these endomorphism by ${\text {End}}_R(V)$.

#### Remark 2

Other equivalent terminologies for *R*-linear maps include *R*-**module homomorphism**, or *R*-**homomorphism** in short.

When considering a free *R*-module $V \cong R^{m}$, every *R*-endomorphism is uniquely represented by an $m \times m$-matrix with entries in *R* and vice versa by applying the conventional matrix multiplication. In particular,

$$\begin{aligned} {\text {End}}_R(R^{m}) \cong {\text {M}}_m(R). \end{aligned}$$

For matrices $A,B \in {\text {M}}_m (R)$, we have for the **determinant** that

$$\begin{aligned} \det (A \cdot B) = \det (A) \cdot \det (B). \end{aligned}$$

Observe that given a matrix $A \in {\text {M}}_m(R)$, we have that *A* is invertible if and only if $\det (A)$ is invertible in *R*.

Another basic result states the relation between the order of the matrix *A*, and the order of its determinant.

#### Lemma 1

Let $$A \in {\text {Tor}}({\text {GL}}_m(R))$$. Then $${\text {ord}}(\det (A)) \mid {\text {ord}}(A).$$

The notions of eigenvectors and eigenvalues remain very similar as in linear algebra: $v \in V$ is an **eigenvector** of $\theta$ if there exists $\lambda \in R$ such that $\theta (v) = \lambda \cdot v$. Here, $\lambda$ is called the **eigenvalue** of *v* under $\theta$. The concept of an eigenbasis is also very similar: $\theta$ has an **eigenbasis** if there exists a basis of *V* consisting of eigenvectors of $\theta$.

### Induced homomorphisms and eigenvectors

Let *R* and *S* be commutative rings with unity, and let $\varphi :R \rightarrow S$ be a ring homomorphism. In particular, $\varphi$ induces on *S* a natural *R*-module structure where we define $r \cdot s:= \varphi (r) \cdot s$ for all $r \in R$ and $s \in S$. Using this *R*-module structure on *S*, the map $\varphi$ is an *R*-linear map. This naturally extends to an *R*-linear map of free modules, which we also denote by $\varphi$:

$$\begin{aligned} \varphi :R^{m} \rightarrow S^{m}, \ (r_0, \dotsc ,r_{m-1}) \mapsto (\varphi (r_0), \dotsc ,\varphi (r_{m-1})). \end{aligned}$$

Observe that $\varphi$ also induces the (ring)-homomorphism of matrices

3 $$\begin{aligned} \overline{\varphi } :{\text {M}}_m(R) \rightarrow {\text {M}}_m(S), \ A = (A_{ij})_{0 \le i,j \le m-1} \mapsto \varphi (A) := (\varphi (A_{ij}))_{0 \le i,j \le m-1}. \end{aligned}$$

The homomorphism of matrices can be interpreted in terms of endomorphisms, meaning that $\overline{\varphi }$ naturally induces a map

$$\begin{aligned} \overline{\varphi } :{\text {End}}_R(R^{m}) \rightarrow {\text {End}}_S(S^{m}), \ \theta \mapsto \overline{\varphi }(\theta ), \end{aligned}$$

satisfying the commutative diagram: 
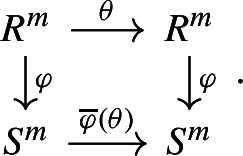
 For $\theta \in {\text {End}}_R(R^{m})$, we denote $\overline{\varphi }(\theta )$ the **induced** *S*-**endomorphism** induced by $\varphi$. Induced endomorphisms behave well with respect to eigenvectors.

#### Lemma 2

Let $v \in R^{m}$ be an eigenvector of $\theta \in {\text {End}}_R(R^{m})$ with eigenvalue $\lambda$. Then $\varphi (v)$ is an eigenvector of $\overline{\varphi }(\theta ) \in {\text {End}}_S(S^{m})$ with eigenvalue $\varphi (\lambda )$.

#### Proof

By commutativity of the above diagram, we get

$$\begin{aligned} \overline{\varphi }(\theta )(\varphi (v)) = \varphi (\theta (v)) = \varphi (\lambda \cdot v) = \varphi (\lambda ) \cdot \varphi (v), \end{aligned}$$

which concludes the proof. $\square$

If $\theta$ admits an eigenbasis in $R^m$, it is not always the case that $\varphi (\theta )$ also admits an eigenbasis in $S^m$. In this paper however, we only consider two types of induced homomorphisms which do preserve the eigenbases.

#### Type I: quotient map of local rings

##### Definition 4

For *R* a local ring with the quotient map $q_R :R \rightarrow R / \mathfrak {m}\cong \mathbb {F}$, we have the isomorphism $V / \mathfrak {m}V \cong \mathbb {F}^{m}$, where $V = R^{m}$. For $\theta \in {\text {End}}_{R}(V)$, we denote the **induced** $\mathbb {F}$-**endomorphism** by $\overline{\theta } \in {\text {End}}_{\mathbb {F}}(V/\mathfrak {m}V)$.

The induced endomorphism $\overline{\theta }$ satisfies the following commutative diagram: 
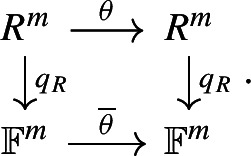


##### Proposition 3

Let *R* be a local ring, and assume that $\theta \in {\text {M}}_m(R)$ has an eigenbasis. Then $\overline{\theta }$ has an eigenbasis over $\mathbb {F}$ in $V/\mathfrak {m}V$.

The proof of Proposition 3 relies on the next two lemmas:

##### Lemma 4

(**Nakayama’s Lemma over local rings**) Let *R* be a local ring, and let *V* be a finitely generated *R*-module. Then any set of generators of *V* over *R* naturally induces a generating set of the $\mathbb {F}$-vector space $V/\mathfrak {m}V$. Conversely, any set of generators of *V* over *R* is induced by a unique basis of $V/\mathfrak {m}V$.

##### Proof

This is a direct consequence of applying local rings to Nakayama’s Lemma [1], which is a well-known result in commutative algebra. $\square$

##### Lemma 5

Let *R* be a local ring with maximal ideal $\mathfrak {m}$ and residue field $\mathbb {F}$, let $V=R^m$ and let $v \in V \setminus \mathfrak {m}V$ be an eigenvector of $\theta \in {\text {M}}_m(R)$. Then $\overline{v}$ is a non-zero eigenvector of $\overline{\theta }$ with eigenvalue $\overline{\lambda } \in \mathbb {F}$.

##### Proof

Since $v \notin \mathfrak {m}V$, we have that ${\overline{v}}$ is non-zero in $V/\mathfrak {m}V$. The rest is an immediate consequence of Lemma 2. $\square$

##### Proof of Proposition 3

This is a direct consequence of Lemmas 4 and 5. $\square$

#### Type II: localization map

##### Definition 5

Let *R* be any commutative ring and let $V = R^m$. For $\mathfrak {p}\in {\text {Spec}}(R)$, we define the localized free $R_{\mathfrak {p}}$-module $R_{\mathfrak {p}}^{m}$ by $V_{\mathfrak {p}}$, where the ring homomorphism $l_{\mathfrak {p}} :R \rightarrow R_{\mathfrak {p}}$ induces the *R*-linear map $l_{\mathfrak {p}} :V \rightarrow V_{\mathfrak {p}}$. For $\theta \in {\text {End}}_{R}(V)$, we denote the **induced** $R_{\mathfrak {p}}$-**endomorphism** by $\theta _{\mathfrak {p}} \in {\text {End}}_{R_{\mathfrak {p}}}(V_{\mathfrak {p}})$.

The induced endomorphism $\theta _{\mathfrak {p}}$ satisfies the following commutative diagram: 
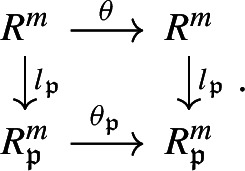


##### Proposition 6

Assume that $\theta \in {\text {M}}_m(R)$ has an eigenbasis, then $\theta _{\mathfrak {p}}$ has an eigenbasis.

We require the following lemma to prove Proposition 6:

##### Lemma 7

Let $\mathfrak {p}\in {\text {Spec}}(R)$, and let $B_V:= \{ v_0, \dotsc ,v_{m-1} \} \subset V$ a basis of *V*, then $B_{V_{\mathfrak {p}}}:= \left\{ \frac{v_0}{1_R}, \dotsc , \frac{v_{m-1}}{1_R} \right\}$ is a basis of $V_{\mathfrak {p}}$.

##### Proof

Let $v_{\mathfrak {p}} = \left( \frac{a_0}{b_0}, \dotsc , \frac{a_{m-1}}{b_{m-1}} \right) \in V_{\mathfrak {p}}$. Define ${\hat{b}}:= \prod _{i=0}^{m-1} b_i$ and ${\hat{b}}_j:= \prod _{0 \le i \le m-1, i \ne j} b_i$, which are elements in $R {\setminus } \mathfrak {p}$ since this set is closed under multiplication. Observe that

$$\begin{aligned} {\hat{b}} \cdot v_{\mathfrak {p}} = \left( \frac{{\hat{b}}_0 \cdot a_0}{1_R}, \dotsc , \frac{{\hat{b}}_{m-1} \cdot a_{m-1}}{1_R} \right) , \end{aligned}$$

which is contained in the image of $l_{\mathfrak {p}}$. Hence there exist $r_0, \dotsc ,r_{m-1} \in R$ such that ${\hat{b}} \cdot v_{\mathfrak {p}} = \sum _{i=0}^{m-1} r_i \cdot \left( \frac{v_i}{1_R} \right)$, which implies that

$$\begin{aligned} v_{\mathfrak {p}} := \sum _{i=0}^{m-1} \frac{r_i}{{\hat{b}}} \cdot \left( \frac{v_i}{1_R} \right) . \end{aligned}$$

Hence $B_{V_{\mathfrak {p}}}$ is a generating set of $V_{\mathfrak {p}}$. Since $B_{V_{\mathfrak {p}}}$ has *m* elements, and $V_{\mathfrak {p}}$ has dimension *m* as a free $R_{\mathfrak {p}}$-module, we conclude that $B_{V_{\mathfrak {p}}}$ is a basis of $V_{\mathfrak {p}}$. $\square$

##### Proof of Proposition 6

By Lemma 2, if $v \in V$ is an eigenvector of $\theta \in {\text {End}}_R(V)$ with eigenvalue $\lambda$, then $l_{\mathfrak {p}}(v)$ is an eigenvector of $\theta _{\mathfrak {p}}$ with eigenvalue $l_{\mathfrak {p}}(\lambda ) = \frac{\lambda }{1_R}$. The claim follows directly from Lemma 7. $\square$

### Useful matrix identities

We show some matrix identities which are useful for studying column parity mixers. These identities are valid for all commutative rings *R* with unity.

#### Definition 6

Let $a = (a_0, \dotsc ,a_{m-1})^{\textsf{T}} \in R^m$ be an *m*-tuple viewed as a column vector. We define the **column matrix** of *a* as the $m \times m$-matrix

$$\begin{aligned} {\text {col}}(a) = \begin{pmatrix} a_0 &  a_0 &  \cdots &  a_0 \\ a_1 &  a_1 &  \cdots &  a_1 \\ \vdots &  \vdots &  \vdots &  \vdots \\ a_{m-1} &  a_{m-1} &  \cdots &  a_{m-1} \end{pmatrix} \in {\text {M}}_m(R). \end{aligned}$$

#### Lemma 8

Consider the vector $b = (b_0, \dotsc ,b_{m-1})^{\textsf{T}} \in R^m$, then

$$\begin{aligned} {\text {col}}(a) \cdot b = \left( \sum _{i=0}^{m-1} b_i \right) \cdot a. \end{aligned}$$

#### Proof

This is a matter of simple verification of matrix multiplication. $\square$

#### Corollary 9

Let $$a,b \in R^m$$, then

$$\begin{aligned} {\text {col}}(a) \cdot {\text {col}}(b) = \left( \sum _{i=0}^{m-1} b_i \right) \cdot {\text {col}}(a). \end{aligned}$$

#### Proposition 10

Let $a \in R^m$. Then for any $t \in \mathbb {Z}_{>0}$, we have that

$$\begin{aligned} {\text {col}}(a)^t = \left( \sum _{i=0}^{m-1} a_i \right) ^{t-1} \cdot {\text {col}}(a). \end{aligned}$$

#### Proof

We use induction on *t*. Let $t = 1$, then

$$\begin{aligned} \left( \sum _{i=0}^{m-1} a_i \right) ^{t-1} \cdot {\text {col}}(a) = \left( \sum _{i=0}^{m-1} a_i \right) ^0 \cdot {\text {col}}(a) = 1_R \cdot {\text {col}}(a) = {\text {col}}(a)^1, \end{aligned}$$

which concludes the first induction step.

Now assume our claim is true for $t = k$ for some $k > 1$. For $t = k+1$, we get

$$\begin{aligned} {\text {col}}(a)^{k+1}&= {\text {col}}(a)^k \cdot {\text {col}}(a) \\&= \left( \sum _{i=0}^{m-1} a_i \right) ^{k-1} \cdot {\text {col}}(a)^2 \\&= \left( \sum _{i=0}^{m-1} a_i \right) ^{k-1} \cdot \left( \sum _{i=0}^{m-1} a_i \right) \cdot {\text {col}}(a) \\&= \left( \sum _{i=0}^{m-1} a_i \right) ^k \cdot {\text {col}}(a), \end{aligned}$$

where the second equality is due to the induction hypothesis, and the third equality due to Lemma 8. This concludes the induction hypothesis, and thus the proof. $\square$

## Circulant rings

Circulant matrices over $\mathbb {F}_2$ are square matrices over $\mathbb {F}_2$ such that every column is a cyclic shift downwards of the column on its left side. As a result, a circulant matrix is fully determined by its first column. The set of $m \times m$-circulant matrices form a ring under matrix addition and multiplication, which is isomorphic to the quotient ring $R_m:= \mathbb {F}_2[X] / \langle X^m - 1 \rangle$. A more detailed discussion about circulant rings can be found in [5]. An equivalent representation of the effect of circulant matrices on vectors in $\mathbb {F}_2^m$, is to induce $\mathbb {F}_2^m$ with an $R_m$-module structure, where scaling a vector $v \in \mathbb {F}_2^m$ by $r \in R_m$ is simply multiplying *v* by the corresponding circulant matrix of *r*. Using this module-theoretic setting, we can deduce properties of circulant matrices from the ring structure of $R_m$ and the module structure of $\mathbb {F}_2^m$.

In this section, we study modules over **circulant rings**, which are generalizations of the ring of circulant matrices. Concretely, circulant rings are defined as follows:

### Definition 7

**Circulant rings** are commutative rings of the form

$$\begin{aligned} R_{m_1, \dotsc ,m_n} := \mathbb {F}_2 [X_1, \dotsc ,X_n] / \big \langle X_1^{m_1} - 1, \dotsc , X_n^{m_n} - 1 \big \rangle . \end{aligned}$$

We denote the set of monomials of $R_{m_1, \dotsc ,m_n}$ as

$$\begin{aligned} M_{m_1, \dotsc ,m_n} := \left\{ \prod _{i=1}^n X_i^{q_i} \mid 0 \le q_i \le m_i - 1 \right\} . \end{aligned}$$

We show that vector space over $\mathbb {F}_2$ of dimension $\prod _{i=1}^n m_i$ admits a natural $R_{m_1, \dotsc ,m_n}$-module structure, which can be considered as a generalization of the notion of circulant rings.

Moreover, we provide an algebraic analysis of local circulant rings. To ease notation, we define the ideals

$$\begin{aligned} \mathfrak {a}_{m_1, \dotsc ,m_n}&:= \big \langle X_1^{m_1} - 1, \dotsc , X_n^{m_n} - 1 \big \rangle , \\ \mathfrak {m}_n&:= \mathfrak {a}_{1, \dotsc ,1} := \langle X_1 - 1, \dotsc , X_n-1 \rangle , \end{aligned}$$

both being ideals of $\mathbb {F}_2[X_1, \dotsc ,X_n]$, and where $\mathfrak {m}_n$ is a maximal ideal.

### Circulant modules: a geometric interpretation

Consider the vector space $V_{m_1, \dotsc ,m_n}$ as the $\mathbb {F}_2$-tensor product

$$\begin{aligned} V_{m_1, \dotsc ,m_n} := \bigotimes _{i=1}^n \mathbb {F}_2^{m_i}, \end{aligned}$$

which has dimension $\prod _{i=1}^n m_i$. We define the standard basis of $V_{m_1, \dotsc ,m_n}$ as

$$\begin{aligned} B_{m_1, \dotsc ,m_n} := \left\{ \otimes _{i=1}^n \textsf{e}_{j_i} \mid 0 \le j_i \le m_i - 1 \hbox { for \,each}\, i \hbox {\,from}\, 1\, to\, n \right\} . \end{aligned}$$

There is a natural $R_{m_1, \dotsc ,m_n}$-module on the vector space $V_{m_1, \dotsc ,m_n}$. To see this, consider the map

$$\begin{aligned} \mu _* :M_{m_1, \dotsc ,m_n} \times B_{m_1, \dotsc ,m_n}&\rightarrow B_{m_1, \dotsc ,m_n} \\ \left( \prod _{i=1}^n X_i^{q_i}, \otimes _{i=1}^n \textsf{e}_{j_i} \right)&\mapsto \otimes _{i=1}^n \textsf{e}_{j_i + q_i \bmod m_i}, \end{aligned}$$

for all $0 \le j_i \le m_i - 1$, which $\mathbb {F}_2$-linearly extends to the map

$$\begin{aligned} \mu :R_{m_1, \dotsc ,m_n} \times V_{m_1, \dotsc ,m_n} \rightarrow V_{m_1, \dotsc ,m_n}. \end{aligned}$$

Note that $V_{m_1, \dotsc ,m_n}$ is an $R_{m_1, \dotsc ,m_n}$-module under $\mu$.

#### Definition 8

The natural $R_{m_1, \dotsc ,m_n}$-action on $V_{m_1, \dotsc ,m_n}^{\omega }$ for some $\omega \in \mathbb {Z}_{>0}$ induced by $\mu$ is called the **circulant module** of rank $\omega$.

#### Proposition 11

A circulant module of rank $\omega$ is a free $R_{m_1, \dotsc ,m_n}$-module of rank $\omega$.

#### Proof

It suffices to show this for circulant modules of rank 1. Consider the following natural 1-to-1 mapping $\vartheta :B_{m_1, \dotsc ,m_n} \rightarrow M_{m_1, \dotsc ,m_n}$ such that

$$\begin{aligned} \vartheta _* :B_{m_1, \dotsc ,m_n} \rightarrow M_{m_1, \dotsc ,m_n}, \ \otimes _{i=1}^n \textsf{e}_{j_i} \mapsto \prod _{i=1}^n X_i^{j_i}, \end{aligned}$$

which linearly extends to the bijective map

$$\begin{aligned} \vartheta :V_{m_1, \dotsc ,m_n} \rightarrow R_{m_1, \dotsc ,m_n}. \end{aligned}$$

This map can be easily verified to be a $R_{m_1, \dotsc ,m_n}$-linear map, hence we have constructed a natural $R_{m_1, \dotsc ,m_n}$-isomorphism. $\square$

#### Remark 3

From the above proposition, we have the commutative diagram 
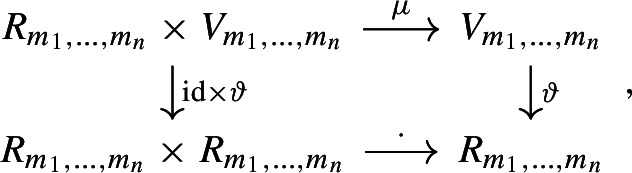


where the dot in the lower row is the natural product operation of the ring $R_{m_1, \dotsc ,m_n}$. The vertical maps are one-to-one correspondences, which implies that the circulant $R_{m_1, \dotsc ,m_n}$-module $V_{m_1, \dotsc ,m_n}$ is indeed free of rank one, with corresponding $R_{m_1, \dotsc ,m_n}$-module isomorphism $\vartheta$.

#### Example 2

We show an example of a circulant module in action. Consider the vector space $V_{16, 64}:= \mathbb {F}_2^{16} \otimes _{\mathbb {F}_2} \mathbb {F}_2^{64}$, and the circulant ring $R_{16,64}$. Consider the vector $v = \textsf{e}_{12} \otimes _{\mathbb {F}_2} \textsf{e}_{55} \in V_{16,64}$, and the polynomial $f:= X^{14} Y^{21} + X^3 Y^9 + 1 \in R_{16,64}$. Observe that $\vartheta (v) = X^{12} Y^{55}$, and so

$$\begin{aligned} f \cdot \vartheta (v)&= (X^{14} Y^{21} + X^3 Y^9 + 1) \cdot X^{12} Y^{55} = X^{26} Y^{76} + X^{15} Y^{64} + X^{12} Y^{55} \\&\equiv X^{10} Y^{12} + X^{15} + X^{12} Y^{55}, \end{aligned}$$

in $R_{16,64}$. From the commutative diagram in Remark 3, we get

$$\begin{aligned} \mu (f,v)&:= \vartheta ^{-1}(f \cdot \vartheta (v)) = \vartheta ^{-1}(X^{10} Y^{12} + X^{15} + X^{12} Y^{55}) \\&= \textsf{e}_{10} \otimes _{\mathbb {F}_2} \textsf{e}_{12} + \textsf{e}_{15} \otimes _{\mathbb {F}_2} \textsf{e}_0 + \textsf{e}_{12} \otimes _{\mathbb {F}_2} \textsf{e}_{55}. \end{aligned}$$

#### Example 3

Consider the ring $R_m = \mathbb {F}_2 [X] / \langle X^m - 1 \rangle$. Let $\textsf{e}_i \in \mathbb {F}_2^m$ and $X^j \in R_m$, where $0 \le i,j < m$. By definition of $\mu$, we have $\mu (X^j, \textsf{e}_i) = \textsf{e}_{i + j}$. Note that this is exactly the same as $\textsf{circ}(\textsf{e}_j) \cdot \textsf{e}_i$, where $\textsf{circ}(\textsf{e}_j)$ refers to the circulant matrix with $\textsf{e}_j$ as its first column. Since this is true for all $0 \le i,j < m$, and since the $X^j$ and $\textsf{e}_i$ span $R_m$ and $\mathbb {F}_2^m$ respectively, one can verify that the circulant $R_m$-module is indeed equivalent to the module induced on $\mathbb {F}_2^m$ over the ring of $m \times m$-circulant matrices over $\mathbb {F}_2$, which was described at the beginning of this section. This justifies the viewpoint that circulant modules can be considered generalizations of the notion of circulant matrices.

#### Example 4

Consider the ring $R_{4,32}$. An interesting case is the free module action of $R_{4,32}$ over $(\mathbb {F}_2^4 \otimes \mathbb {F}_2^{32})^3$. By Proposition 11, this module is isomorphic to the free module $R_{4,32}^3$, which is in particular useful for studying the linear layer of Xoodoo, which we cover in Sect. 5.

### Classification of local circulant rings

We prove that a circulant ring $R_{m_1, \dotsc ,m_n}$ is a local ring if and only if $m_i$ is a power of 2 for all $1 \le i \le m$.

#### Lemma 12

The ideal $\mathfrak {m}_{n}$ is the unique maximal ideal containing the ideal $\mathfrak {a}_{2^{l_1}, \dotsc ,2^{l_n}}$, where $l_1, \dotsc ,l_n \in \mathbb {Z}_{\ge 0}$.

#### Proof

Since $\mathbb {F}_2[X_1, \dotsc ,X_n]$ has characteristic 2, we have that

4 $$\begin{aligned} X^{2^{l_i}}-1 = (X-1)^{2^{l_i}}, \end{aligned}$$

for each $1 \le i \le n$, which immediately implies that $\mathfrak {a}_{2^{l_1}, \dotsc ,2^{l_n}} \subseteq \mathfrak {m}_n$. Note that (4) also implies that $\mathfrak {m}_n$ is contained in the radical of $\mathfrak {a}_{2^{l_1}, \dotsc ,2^{l_n}}$, which in turn implies that $\mathfrak {m}_n = r(\mathfrak {a}_{2^{l_1}, \dotsc ,2^{l_n}})$. Since every maximal ideal containing $\mathfrak {a}_{2^{l_1}, \dotsc ,2^{l_n}}$ must contain $r(\mathfrak {a}_{2^{l_1}, \dotsc ,2^{l_n}})$, it must also contain $\mathfrak {m}_n$. But $\mathfrak {m}_n$ is already maximal, hence uniqueness is proven. $\square$

#### Theorem 13

A circulant ring $R_{m_1, \dotsc ,m_n}$ is a local ring if and only if $m_i$ is a power of 2 for all $1 \le i \le m$.

#### Proof

$(\Leftarrow )$ - Assume that $m_i$ is of the form $2^{l_i}$, where $l_i \in \mathbb {Z}_{\ge 0}$ for all $1 \le i \le n$. Since $\mathfrak {m}_n$ is the unique maximal ideal containing $\mathfrak {a}_{2^{l_1}, \dotsc ,2^{l_n}}$ by the above lemma, we have that $\overline{\mathfrak {m}}_n$ must be the unique maximal ideal in $R_{2^{l_1}, \dotsc ,2^{l_n}}$ as shown in [6]. This shows that $R_{2^{l_1}, \dotsc ,2^{l_n}}$ is local.

$(\Rightarrow )$ - Assume that there exists $m_j$ for some $1 \le j \le n$ such that $m_j$ is not a power of 2. We may assume without loss of generality that $m_1$ is not a power of 2. Consider the ideal $\mathfrak {m}':= \langle \Phi _{m_1} (X_1), X_2 - 1, \dotsc , X_n - 1 \rangle$ where $\Phi _{m_1}$ is the $m_1$-th cyclotomic polynomial. Note that $\Phi _{m_1}$ has degree larger than 1, since $m_1$ is not a power of 2. By the third isomorphism theorem for rings, we get

$$\begin{aligned} R_{m_1, \dotsc ,m_n} / \overline{\mathfrak {m}}'&= (\mathbb {F}_2[X_1, \dotsc ,X_n] / \mathfrak {a}_{m_1, \dotsc ,m_n} ) / (\mathfrak {m}' / \mathfrak {a}_{m_1, \dotsc ,m_n} ) \\&\cong \mathbb {F}_2[X_1, \dotsc ,X_n] / \mathfrak {m}' \cong \mathbb {F}_2[X_1] / \Phi _{m_1}(X_1). \end{aligned}$$

Since $\Phi _{m_1}$ is irreducible in $\mathbb {F}_2[X]$, we have that $\mathbb {F}_2[X_1] / \Phi _{m_1}(X_1)$ is a field isomorphic to ${\text {GF}}(2^{\deg (\Phi _{m_1})})$. Hence $\overline{\mathfrak {m}}'$ is a maximal ideal of $R_{m_1, \dotsc ,m_n}$ which is not equal to $\mathfrak {m}_n$, thus $R_{m_1, \dotsc ,m_n}$ is not a local ring. This concludes the proof. $\square$

#### Note 14

For the remainder of this section, we denote $\overline{\mathfrak {m}}_n$ simply by $\mathfrak {m}_n$, which will not cause confusion due to uniqueness of $\mathfrak {m}_n$.

#### Lemma 15

The residue field of a local circulant ring *R* is isomorphic to $\mathbb {F}_2$, with quotient map

$$\begin{aligned} q_R :R \rightarrow \mathbb {F}_2, \ f \mapsto f(1^n). \end{aligned}$$

#### Proof

By the third isomorphism theorem for rings, we get

$$\begin{aligned} R_{2^{l_1}, \dotsc ,2^{l_n}} / \overline{\mathfrak {m}}_n&= (\mathbb {F}_2[X_1, \dotsc ,X_n] / \mathfrak {a}_{2^{l_1}, \dotsc ,2^{l_n}}) / (\mathfrak {m}_n / \mathfrak {a}_{2^{l_1}, \dotsc ,2^{l_n}}) \\&\cong \mathbb {F}_2[X_1, \dotsc ,X_n] / \mathfrak {m}_n \cong \mathbb {F}_2, \end{aligned}$$

where by construction the last isomorphism is indeed the map $f \mapsto f(1^n)$. $\square$

#### Remark 4

For $f \in R$, we denote $q_R(f)$ by $\overline{f}$.

#### Corollary 16

For *R* a local circulant ring, we have that $f \in R$ is invertible if and only if $f(1^n) \ne 0$.

#### Proof

Since *R* is a local ring, we have that *f* is invertible if and only if $f \notin \mathfrak {m}_n$. This is indeed equivalent to $f(1^n) \ne 0$ by the above lemma. $\square$

#### Corollary 17

For *R* a local circulant ring, we have that $f \in R$ is invertible if and only if *f* contains an odd number of terms.

#### Proof

By the above corollary, we conclude that every term is invertible. If *f* has *t* terms, then $f(1^n) \equiv t \bmod 2$ which is not equal to 0 if and only if *t* is odd. This concludes the proof. $\square$

### General linear group over local circulant rings

Let $R:= R_{2^{l_1}, \dotsc ,2^{l_n}}$ be a local circulant ring, and consider $q_R$ as in Lemma 15. This map can be extended to the map of $m \times m$-matrices

$$\begin{aligned} q_{m,R} :{\text {M}}_m(R) \rightarrow {\text {M}}_m(\mathbb {F}_2), \ A := (A_{ij})_{0 \le i,j \le m-1} \mapsto (\overline{A_{ij}})_{0 \le i,j \le m-1}. \end{aligned}$$

#### Remark 5

Just as for the case of $q_R$, for $A \in {\text {M}}_m(\mathbb {F}_2)$, we denote $q_{m,R}(A)$ by $\overline{A}$.

Observe that $\det (\overline{A}) = \overline{\det (A)}$, since the expression of the determinant consists of finite sums of finite products of entries of *A*, which split under $q_R$. This implies that $q_{m,R}$ maps ${\text {GL}}_m(R)$ to ${\text {GL}}_m(\mathbb {F}_2)$. Moreover, we have that the preimage of ${\text {GL}}_m(\mathbb {F}_2)$ under $q_{m,R}$ is exactly ${\text {GL}}_m(R)$, as a result of the following lemma:

#### Lemma 18

Let $A \in {\text {M}}_m(R)$. Then $A \in {\text {GL}}_m(R)$ if and only if $\overline{A} \in {\text {GL}}_m(\mathbb {F}_2)$.

#### Proof

Due to locality of *R*, we have the following equivalent statements:

$$\begin{aligned} A \in {\text {GL}}_m(R)&\Leftrightarrow \det (A) \in R^* \Leftrightarrow \det (A) \notin \mathfrak {m}_n \Leftrightarrow \det (\overline{A}) \in \mathbb {F}_2^* \Leftrightarrow \overline{A} \in {\text {GL}}_m(\mathbb {F}_2). \end{aligned}$$

$$\square $$

The above lemma implies that $q_{m,R} \mid _{{\text {GL}}_m(R)} :{\text {GL}}_m(R) \rightarrow {\text {GL}}_m(\mathbb {F}_2)$ is a surjective group homomorphism. Let us denote $q_{m,R} \mid _{{\text {GL}}_m(R)}$ by $q_{m,R}^*$. From Lemma 18, we conclude that

$$\begin{aligned} \ker (q_{m,R}^*) = \{ I_m + A : A \in {\text {M}}_m(\mathfrak {m}_n) \}. \end{aligned}$$

This implies that

5 $$\begin{aligned} \# \ker (q_{m,R}^*) = \# {\text {M}}_m(\mathfrak {m}_n) = (\# \mathfrak {m}_n)^{m^2} = \left( 2^{( \prod _{i=1}^n 2^{l_i} ) - 1} \right) ^{m^2}, \end{aligned}$$

which in particular means that the order of the group $\ker (q_{m,R}^*)$ is a power of 2. By Lagrange’s theorem, the order of an element $I_m + A \in \ker (q_{m,R}^*)$, where $A \in {\text {M}}_m(\mathfrak {m}_n)$, is of the form $2^{\lambda }$ where $\lambda \in \mathbb {Z}_{\ge 0}$. Note that

$$\begin{aligned} (I_m +A)^{2^{\lambda }} = I_m + A^{2^{\lambda }}, \end{aligned}$$

by the binomial theorem of Newton, and since *R* is of characteristic 2. Hence ${\text {ord}}(I_m + A)$ is the smallest number of the form $2^{\lambda }$ such that $A^{2^{\lambda }} = 0_{m \times m}$. In particular, *A* is a **nilpotent matrix**.

#### Lemma 19

Let $A \in {\text {M}}_m(\mathfrak {m}_n)$ and define $l = \max (l_i: 1 \le i \le n)$. Then we have $A^{n \cdot 2^l} = 0_{m \times m}$.

#### Proof

In this proof, we use $\mathbb {Z}_{\ge 0}^n$ as an index set, and we let $\textsf{e}_i$ be the *i*-th unit vector in $\mathbb {Z}_{\ge 0}^n$ where $0 \le i \le n-1$.

By assumption of the lemma, there exist matrices $A_{\textsf{e}_i} \in {\text {M}}_m(R)$ such that

6 $$\begin{aligned} A = \sum _{i=1}^n (X_i - 1) \cdot A_{\textsf{e}_i}. \end{aligned}$$

From this, we can construct matrices $A_{j_1 \textsf{e}_1 + \cdots + j_n \textsf{e}_n} \in {\text {M}}_m(R)$ such that

7 $$\begin{aligned} A^2 = \sum _{\begin{array}{c} 0 \le j_1, \dotsc ,j_n \le 2 \\ j_1 + \cdots + j_n = 2 \end{array}} A_{j_1 \textsf{e}_1 + \cdots + j_n \textsf{e}_n} \cdot \prod _{i=1}^n (X_i - 1)^{j_i}, \end{aligned}$$

where

$$\begin{aligned} A_{j_1 \textsf{e}_1 + \cdots + j_n \textsf{e}_n} = \sum _{\begin{array}{c} 0 \le j_1, \dotsc ,j_n \le 2 \\ j_1 + \cdots + j_n = 2 \end{array}} A_{\textsf{e}_i}^{j_i}. \end{aligned}$$

Note that the matrices $A_{\textsf{e}_i}$ satisfying (6) are not unique. For the proof, it suffices to only knowing its existence.

By inductively applying this reasoning, one can show that for all $k \in \mathbb {Z}_{>0}$, there exists a family of matrices $A_{j_1 \textsf{e}_1 + \cdots + j_n \textsf{e}_n} \in {\text {M}}_m(R)$ where $j_1 + \cdots + j_n = k$ such that

8 $$\begin{aligned} A^k = \sum _{\begin{array}{c} 0 \le j_1, \dotsc ,j_n \le k \\ j_1 + \cdots + j_n = k \end{array}} A_{j_1 \textsf{e}_1 + \cdots + j_n \textsf{e}_n} \cdot \prod _{i=1}^n (X_i - 1)^{j_i}. \end{aligned}$$

When $k \ge n \cdot 2^l$, we must have that $j_i \ge 2^{l_i}$ for some *i*, which implies that $\prod _{i=1}^n (X_i - 1)^{j_i} \in \mathfrak {m}_n$ for all $j_1, \dotsc ,j_n$ satisfying $j_1 + \cdots + j_n = k$. Hence we have $A^{n \cdot 2^l} = 0_{m \times m} \in {\text {M}}_m(R)$ by applying Eq. (8), which concludes the proof. $\square$

#### Corollary 20

For $B \in \ker (q_{m,R}^*)$, we have that ${\text {ord}}(B) \mid 2^{l + \lceil \log _2(n) \rceil }$.

#### Proof

Every element $B \in \ker (q_{m,R}^*)$ is of the form $I_m + C$, where $C \in {\text {M}}_m(\mathfrak {m}_n)$. Observe that

$$\begin{aligned} 2^{l + \lceil \log _2(n) \rceil } = 2^{\lceil \log _2(n) \rceil } \cdot 2^l \ge n \cdot 2^l. \end{aligned}$$

From this identity together with Lemma 19, we have

$$\begin{aligned} B^{2^{l + \lceil \log _2(n) \rceil }}&= (I_m + C)^{2^{l + \lceil \log _2(n) \rceil }} = I_m^{2^{l + \lceil \log _2(n) \rceil }} + C^{2^{l + \lceil \log _2(n) \rceil }} = I_m + 0_{m \times m} \\&= I_m, \end{aligned}$$

which concludes the proof. $\square$

## Column parity mixers

Using circulant modules covered in Sect. 3, we introduce a new approach to column parity mixers (CPMs) which is a vast generalization of circulant column parity mixers (CCPMs) defined in [9].

### Definition 9

Let *R* be a commutative ring with unity. A **column parity mixer** $\theta _z$ (or CPM for short) over *R* of dimension *m* where $z = (z_0, \dotsc ,z_{m-1})^{\textsf{T}} \in R^m$, is an *R*-endomorphism over $R^m$ represented by the matrix

$$\begin{aligned} \theta _z = I_m + {\text {col}}(z) = \begin{pmatrix} 1_R + z_0 &  z_0 &  z_0 &  \cdots &  z_0 \\ z_1 &  1_R + z_1 &  z_1 &  \cdots &  z_1 \\ z_2 &  z_2 &  1_R + z_2 &  \cdots &  z_2 \\ \vdots &  \vdots &  \vdots &  \ddots &  \vdots \\ z_{m-1} &  z_{m-1} &  z_{m-1} &  \cdots &  1_R + z_{m-1} \end{pmatrix} . \end{aligned}$$

We say that *z* is the **parity-folding matrix array**, and $z_0, \dotsc ,z_{m-1}$ are the **parity-folding matrices** of $\theta _z$. The set of all CPMs over *R* of dimension *m* is denoted by ${\text {CPM}}_m(R)$.

A CPM over a circulant ring *R* is called a **circulant column parity mixer**, or CCPM for short.

### Remark 6

Although the terminology might suggest otherwise, parity folding matrices are not necessarily matrices, but rather elements in the ring *R*. The reason for this choice of terminology is that the analogue of parity-folding matrices in the original definition of CPMs in [9] are actually matrices. These two notions coincide in the case when *R* is the ring of circulant matrices.

### Remark 7

The CPMs in Definition 9 are closely related to, but not a full generalization of CPMs introduced in [9], which we refer to for now as the original CPMs. This is because the parity folding matrices in the original CPMs admit all matrices in the matrix ring, which do not form a commutative ring.

Definition 9 is however a generalization of the original CCPMs. In the special case where we consider CCPMs of Definition 9, and $z_0 = z_1 = \cdots = z_{m-1}$, we obtain the original CCPMs, where they use only one parity folding matrix.

### Characteristic polynomial and determinant

We give an expression of the characteristic polynomial and the determinant of a CPM in terms of its parity-folding matrices. We assume for the remainder of this subsection that $\theta _z \in {\text {CPM}}_m(R)$ for some $m \in \mathbb {Z}_{>0}$ and commutative ring *R*.

#### Theorem 21

The characteristic polynomial $p_{\theta _z}(\lambda )$ of $\theta _z$ is

9 $$\begin{aligned} p_{\theta _z}(\lambda ) = \left( \left( 1_R + \sum _{i=0}^{m-1} z_i \right) - \lambda \right) \cdot (1_R - \lambda )^{m-1}. \end{aligned}$$

#### Proof

By definition, $p_{\theta _z}(\lambda ):= \det (\theta _z - \lambda \cdot I_m)$. To compute the determinant of $\theta _z - \lambda \cdot I_m$, we use the property that adding up rows (or columns) to **other** rows (or columns) will not affect the determinant. By adding the first column vector to all the other column vectors of $\theta _z$, followed by adding up all the row vectors from the second till the last row vector to the first row vector, we get

$$\begin{aligned} \det (\theta _z - \lambda \cdot I_m)&= \begin{vmatrix} 1_R + z_0 - \lambda&z_0&z_0&\cdots&z_0 \\ z_1&1_R + z_1 - \lambda&z_1&\cdots&z_1 \\ z_2&z_2&1_R + z_2 - \lambda&\cdots&z_2 \\ \vdots&\vdots&\vdots&\ddots&\vdots \\ z_{m-1}&z_{m-1}&z_{m-1}&\cdots&1_R + z_{m-1} - \lambda \end{vmatrix} \\&= \begin{vmatrix} 1_R + z_0 - \lambda&\lambda - 1_R&\lambda - 1_R&\cdots&\lambda - 1_R \\ z_1&1_R - \lambda&0&\cdots&0 \\ z_2&0&1_R - \lambda&\cdots&0 \\ \vdots&\vdots&\vdots&\ddots&\vdots \\ z_{m-1}&0&0&\cdots&1_R - \lambda \end{vmatrix} \\&= \begin{vmatrix} 1_R + \left( \sum _{i=0}^{m-1}z_i \right) - \lambda&0&0&\cdots&0 \\ z_1&1_R - \lambda&0&\cdots&0 \\ z_2&0&1_R - \lambda&\cdots&0 \\ \vdots&\vdots&\vdots&\ddots&\vdots \\ z_{m-1}&0&0&\cdots&1_R - \lambda \end{vmatrix}. \end{aligned}$$

Denote the last matrix by *A* and denote $A_{(i,j)}$ as the $m-1 \times m-1$-matrix by removing the *i*-th row and the *j*-th column of *A*. Then

10 $$\begin{aligned} \det (\theta _z - \lambda \cdot I_m)&= \det (A) \nonumber \\&= \sum _{j=0}^{m-1} (-1)^{j} \cdot A_{0,j} \cdot \det (A_{(0,j)}) \nonumber \\&= A_{0,0} \cdot \det (A_{(0.0)}) + \sum _{j=1}^{m-1} (-1)^{j} \cdot A_{0,j} \cdot \det (A_{(0,j)}) \nonumber \\&= A_{0,0} \cdot \det (A_{(0.0)}), \end{aligned}$$

where the last equation holds because $A_{0,j} = 0$ for $j > 0$. Observe that $A_{0,0} = \left( 1_R + \sum _{i=0}^{m-1} z_i \right) - \lambda$ and $A_{(0,0)} = (1_R - \lambda ) \cdot I_{m-1}$, the latter implying that $\det (A_{(0,0)}) = (1_R - \lambda )^{m-1}$. Substituting these values in (10), we obtain

$$\begin{aligned} \det (\theta _z - \lambda \cdot I_m) = A_{0,0} \cdot \det (A_{(0.0)}) = \left( \left( 1_R + \sum _{i=0}^{m-1} z_i \right) - \lambda \right) \cdot (1_R - \lambda )^{m-1}, \end{aligned}$$

which concludes the proof. $\square$

#### Corollary 22

The determinant of $\theta _z$ equals

$$\begin{aligned} \det (\theta _z) = 1_R + \sum _{i=0}^{m-1} z_i. \end{aligned}$$

#### Proof

The determinant equals the constant term of the characteristic polynomial of $\theta _z$, which from Eq. (9) equals $1_R + \sum _{i=0}^{m-1} z_i$. $\square$

#### Note 23

By the above corollary, $p_{\theta _z}(\lambda )$ can be expressed as

11 $$\begin{aligned} p_{\theta _z}(\lambda ) = (\det (\theta _z) - \lambda ) \cdot (1_R - \lambda )^{m-1}. \end{aligned}$$

We will use this expression for the remainder of this paper.

### Eigenvectors and eigenspaces

#### Lemma 24

Define

$$\begin{aligned} E_1 := \left\{ v=(v_0, \dotsc ,v_{m-1})^{\textsf{T}} : \sum _{i=0}^{m-1} v_i = 0 \right\} . \end{aligned}$$

Then all elements in $E_1$ have eigenvalue $1_R$, and $E_1$ is a free *R*-module of rank $m-1$.

#### Proof

Observe that for all $v \in E_1$, we have

$$\begin{aligned} \theta _z(v) = (I_m + {\text {col}}(z)) \cdot v = v + \left( \sum _{i=0}^{m-1} v_i \right) \cdot z = v + 0 \cdot v = v, \end{aligned}$$

which proves that all elements in $E_1$ have eigenvalue $1_R$. Observe that $E_1$ has $m-1$ degrees of freedom, since every $m-1$-tuple of elements in *R* uniquely determines an element in $E_1$. Hence $E_1$ is a free *R*-module of rank $m-1$. $\square$

#### Lemma 25

The vector $z = (z_0, \dotsc ,z_{m-1})^{\textsf{T}} \in V$ is an eigenvector of $\theta _z$ with eigenvalue $\det (\theta _z)$.

#### Proof

Observe that

$$\begin{aligned} \theta _z(z)&= (I_m + {\text {col}}(z)) \cdot z = z + {\text {col}}(z) \cdot z = z + \left( \sum _{i=0}^{m-1} z_i \right) \cdot z = \left( 1_R + \sum _{i=0}^{m-1} z_i \right) \cdot z \\&= \det (\theta _z) \cdot z, \end{aligned}$$

which finishes the proof. $\square$

#### Lemma 26

Assume that $\det (\theta _z) - 1_R$ is invertible in *R*, and define

$$\begin{aligned} E_2 = \left\{ r \cdot z : r \in R \right\} . \end{aligned}$$

Then $E_2$ is a free *R*-submodule of rank 1, and $E_1 \cap E_2 = \{ 0 \}$.

#### Proof

Observe that for all $r_1, r_2 \in R$ such that $(r_1 - r_2) \cdot z = 0_m$, we have that $(r_1 - r_2) \cdot \left( \sum _{i=0}^{m-1} z_i \right) = 0$. Since $\sum _{i=0}^{m-1} z_i = \det (\theta _z) - 1_R$ is invertible, it must be true that $r_1 = r_2$. This shows that $E_2$ is a free *R*-submodule of rank 1.

Let $x \in E_2$, then there exists $r_x \in R$ such that $x = r_x \cdot z \in E_2$. Note that

12 $$\begin{aligned} x = r_x \cdot z \in E_1 \Longleftrightarrow \sum _{i=0}^{m-1} r_x \cdot z_i := r_x \cdot \left( \sum _{i=0}^{m-1} z_i \right) := r_x \cdot (\det (\theta _z) - 1_R) = 0. \end{aligned}$$

Since by our assumption $\det (\theta _z) - 1_R$ is invertible in *R*, Eq. (12) holds if and only if $r_x = 0$, which implies that $x \in E_1$ if and only if $x = 0$. This implies that $E_1 \cap E_2 = \{ 0 \}$, which concludes the proof. $\square$

#### Proposition 27

Assume that $\det (\theta _z) - 1_R$ is invertible in *R*, then *V* is a direct sum of eigenspaces $E_1$ and $E_2$ of $\theta _z$ with eigenvalues $1_R$ and $\det (\theta _z)$ respectively.

#### Proof

This is immediate from Lemmas 24, 25 and 26. $\square$

#### Theorem 28

Assume that $\det (\theta _z) - 1_R$ is not invertible, then $\theta _z$ does not have an eigenbasis.

#### Proof

Since $\det (\theta _z) - 1_R$ is not invertible in *R*, there exists a maximal ideal $\mathfrak {m}\in {\text {MaxSpec}}(R)$ such that $\det (\theta _z) - 1_R \in \mathfrak {m}$. In particular, $\det (\theta _z) \equiv 1_R \bmod \mathfrak {m}$.

Assume to the contrary that $\theta _z$ has an eigenbasis. Then by Proposition 6, the induced $R_{\mathfrak {m}}$-endomorphism

$$\begin{aligned} (\theta _z)_{\mathfrak {m}} :V_{\mathfrak {m}} \rightarrow V_{\mathfrak {m}}, \end{aligned}$$

also has an eigenbasis.

Define the field $\mathbb {F}_{\mathfrak {m}}:= R_{\mathfrak {m}} / \mathfrak {m}R_{\mathfrak {m}}$ (this is a field because $R_{\mathfrak {m}}$ is a local ring) and consider the $\mathbb {F}_{\mathfrak {m}}$-module $V_{\mathfrak {m}} / (\mathfrak {m}R_{\mathfrak {m}}) V_{\mathfrak {m}}$. Note that $V_{\mathfrak {m}} / (\mathfrak {m}R_{\mathfrak {m}}) V_{\mathfrak {m}}$ is an *m*-dimensional vector space over $\mathbb {F}_{\mathfrak {m}}$, which implies that $V_{\mathfrak {m}} / (\mathfrak {m}R_{\mathfrak {m}}) V_{\mathfrak {m}} \cong \mathbb {F}_{\mathfrak {m}}^m$. By Proposition 3, the vector space $\mathbb {F}_{\mathfrak {m}}^m$ has an eigenbasis of the induced map $\overline{(\theta _z)_{\mathfrak {m}}} :\mathbb {F}_{\mathfrak {m}}^m \rightarrow \mathbb {F}_{\mathfrak {m}}^m$. Since $\mathbb {F}_{\mathfrak {m}}:= R_{\mathfrak {m}} / \mathfrak {m}R_{\mathfrak {m}} \cong R / \mathfrak {m}$, the corresponding matrix of $\overline{(\theta _z)_{\mathfrak {m}}}$ is the matrix of $\theta _z$ where all entries are taken modulo $\mathfrak {m}$. For this reason, the characteristic polynomial of $\overline{(\theta _z)_{\mathfrak {m}}}$ is the polynomial

13 $$\begin{aligned} p_{\overline{(\theta _z)_{\mathfrak {m}}}}(\lambda ) = \left( \overline{\det (\theta _z)} - \lambda \right) \cdot (1 - \lambda )^{m-1}. \end{aligned}$$

Since $\det (\theta _z) \equiv 1_R \bmod \mathfrak {m}$, we have that $\overline{\det (\theta _z)} = 1$ which implies that the only eigenvalue of $\overline{(\theta _z)_{\mathfrak {m}}}$ is 1. Let $\overline{E}_1$ be the eigenspace of $\overline{(\theta _z)_{\mathfrak {m}}}$ with eigenvalue 1. By standard linear algebra over fields, we get

$$\begin{aligned} \overline{E}_1 := \ker \left( \overline{(\theta _z)_{\mathfrak {m}}} - I_m \right) = \ker (I_m + {\text {col}}(\overline{z}) - I_m) = \ker ({\text {col}}(\overline{z})). \end{aligned}$$

Note that $\dim (\overline{E}_1) = m-1$ since ${\text {col}}(\overline{z})$ has rank 1. But then

$$\begin{aligned} \dim \left( \overline{E}_1 \right) < \dim (V_{\mathfrak {m}} / (\mathfrak {m}R_{\mathfrak {m}}) V_{\mathfrak {m}}) = m, \end{aligned}$$

which means that $\overline{E}_1$ is not an eigenbasis of $\overline{(\theta _z)_{\mathfrak {m}}}$. This contradicts our assumption, hence $\theta _z$ does not have an eigenbasis. $\square$

### Group of invertible column parity mixers

#### Lemma 29

Let $$\theta _z, \theta _{z'} \in {\text {CPM}}_m(R)$$, then

$$\begin{aligned} \theta _z \cdot \theta _{z'} = \theta _{z' + \det (\theta _{z'}) z} \in {\text {CPM}}_m(R), \end{aligned}$$

which in particular implies that ${\text {CPM}}_m(R)$ is closed under multiplication.

#### Proof

This is due to the following:

$$\begin{aligned} \theta _z \cdot \theta _{z'}&= (I_m + {\text {col}}(z)) \cdot (I_m + {\text {col}}(z'))\\&= I_m + {\text {col}}(z) + {\text {col}}(z') + {\text {col}}(z) \cdot {\text {col}}(z') \\&= I_m + {\text {col}}(z) + {\text {col}}(z') + \left( \sum _{i=0}^{m-1} z'_i \right) \cdot {\text {col}}(z) \\&= I_m + {\text {col}}\left( z' + \left( 1_R + \sum _{i=0}^{m-1} z'_i \right) \cdot z \right) \\&= I_m + {\text {col}}(z' + \det (\theta _{z'}) \cdot z ), \end{aligned}$$

where the third equation is due to Corollary 9. $\square$

#### Lemma 30

Let $\theta _z \in {\text {CPM}}_m(R)$ be invertible, then

$$\begin{aligned} \theta _z^{-1} = \theta _{- z \cdot \det (\theta _{z})^{-1}} \in {\text {CPM}}_m(R). \end{aligned}$$

#### Proof

Since $\theta _z$ is invertible, we have that $\det (\theta _z)$ is invertible in *R*, hence $\det (\theta _z)^{-1}$ is well-defined. Then

$$\begin{aligned} \theta _{z'} \cdot \theta _z = I_m \Longleftrightarrow z + \det (\theta _{z}) \cdot z' = 0 \Longleftrightarrow z' = -z \cdot \det (\theta _{z})^{-1}, \end{aligned}$$

which concludes the proof. $\square$

#### Proposition 31

The set ${\text {CPM}}^*_m(R)$ consisting of all invertible CPMs forms a subgroup of ${\text {GL}}_m(R)$.

#### Proof

By Lemma 29, ${\text {CPM}}^*_m(R)$ is closed under multiplication. Moreover, the inverse of a CPM is also a CPM by Lemma 30. This implies that ${\text {CPM}}^*_m(R)$ is indeed a subgroup of ${\text {GL}}_m(R)$. $\square$

#### Lemma 32

Let *R* be a ring of prime characteristic *p*, and let $\theta _z \in {\text {CPM}}^*_m(R)$ such that $\det (\theta _z) = 1_R$ and $\theta _z \ne I_m$. Then ${\text {ord}}(\theta _z) = p$.

#### Proof

Observe that

$$\begin{aligned} \theta _z^p = (I_m + {\text {col}}(z))^p = I_m^p + {\text {col}}(z)^p = I_m + \left( \sum _{i=0}^{m-1} z_i \right) ^{p-1} \cdot {\text {col}}(z), \end{aligned}$$

where the second equation is due to Newton’s Binomial Theorem combined with the fact that all multiples of *p* vanish in rings of characteristic *p*, and where the third equation is due to the identity in Proposition 10. Since $\det (\theta _z) = 1_R$, we have that $\sum _{i=0}^{m-1} z_i = 0$, which implies that $\theta _z^p = I_m$. This means that ${\text {ord}}(\theta _z) \mid p$, which implies that ${\text {ord}}(\theta _z)$ equals either 1 or *p* since *p* is prime. Because $\theta _z \ne I_m$, we have ${\text {ord}}(\theta _z) \ne 1$, which means ${\text {ord}}(\theta _z) = p$. $\square$

#### Lemma 33

Let *R* be a ring of prime characteristic *p*, and let $\theta _z \in {\text {CPM}}^*_m(R)$. Then ${\text {ord}}(\theta _z)$ is either ${\text {ord}}(\det (\theta _z))$ or $p \cdot {\text {ord}}(\det (\theta _z))$.

#### Proof

From Lemma 1, we have that

$$\begin{aligned} {\text {ord}}(\det (\theta _z)) \mid {\text {ord}}(\theta _z). \end{aligned}$$

Note that

$$\begin{aligned} {\text {ord}}(\theta _z) = {\text {ord}}(\det (\theta _z)) \cdot {\text {ord}}\left( \theta _z^{{\text {ord}}(\det (\theta _z))} \right) . \end{aligned}$$

Assuming ${\text {ord}}(\det (\theta _z)) < \infty$, we get

$$\begin{aligned} \det \left( \theta _z^{{\text {ord}}(\det (\theta _z))} \right) = \det (\theta _z)^{{\text {ord}}(\det (\theta _z))} = 1_R. \end{aligned}$$

Hence by Lemma  32, ${\text {ord}}\left( \theta _z^{{\text {ord}}(\det (\theta _z))} \right)$ is either 1 or *p*, which concludes the proof. $\square$

#### Remark 8

The above lemma implies that $\theta _z \in {\text {Tor}}({\text {CPM}}^*_m(R))$ if and only if $\det (\theta _z) \in {\text {Tor}}(R^*)$.

#### Proposition 34

Let $\theta _z \in {\text {CPM}}^*_m(R)$ such that $\det (\theta _z) - 1_R \in R^*$. Then

$$\begin{aligned} {\text {ord}}(\theta _z) = {\text {ord}}(\det (\theta _z)). \end{aligned}$$

#### Proof

By Proposition 27, $\theta _z$ admits an eigenbasis with eigenvalues $\lambda _1 = 1_R$ and $\lambda _2 = \det (\theta _z)$. From this, we conclude that

$$\begin{aligned} {\text {ord}}(\theta _z) = {\text {lcm}}({\text {ord}}(\lambda _1), {\text {ord}}(\lambda _2)) = {\text {lcm}}(1, {\text {ord}}(\det (\theta _z))) = {\text {ord}}(\det (\theta _z)), \end{aligned}$$

which completes the proof. $\square$

We conclude this section by briefly considering CPMs over $\mathbb {F}_2$ and over local circulant rings.

#### Lemma 35

Let $\theta _z \in {\text {CPM}}_m^*(\mathbb {F}_2)$ such that $\theta \ne I_m$. Then ${\text {ord}}(\theta _z) = 2$.

#### Proof

By Lemma 33, we have that ${\text {ord}}(\theta _z)$ is either equal to ${\text {ord}}(\det (\theta _z))$ or $2 \cdot {\text {ord}}( \det (\theta _z) )$. Since $\theta _z$ is invertible, we know that $\det (\theta _z) \in \mathbb {F}_2^*$, which means that $\det (\theta _z) = 1_{\mathbb {F}_2}$. Hence ${\text {ord}}(\det (\theta _z)) = 1$, which means that ${\text {ord}}(\theta _z)$ is either 1 or 2. Since $\theta _z \ne I_m$, we must have that ${\text {ord}}(\theta _z) = 2$, which completes the proof. $\square$

#### Proposition 36

Let $R = R_{2^{l_1}, \dotsc ,2^{l_n}}$ be a local circulant ring, and define $l = \max (l_i: 1 \le i \le n)$. Then for $\theta _z \in {\text {CPM}}_m^*(R)$, we have that ${\text {ord}}(\theta _z) \mid 2^{l+2}$.

#### Proof

$q^*_{m,R}$ restricted to ${\text {CPM}}_m^*(R)$ induces a surjective map to ${\text {CPM}}_m^*(\mathbb {F}_2)$.

Let us first consider the case that $\theta _z \in \ker (q^*_{m,R})$. Since $\theta _z = I_m + {\text {col}}(z)$, we have that $z_0, \dotsc ,z_{m-1} \in \mathfrak {m}_n$. Observe that

$$\begin{aligned} \theta _z^{2^{l + 1}} = (I_m + {\text {col}}(z))^{2^{l+1}} = I_m + {\text {col}}(z)^{2^{1+1}} = I_m + \left( \sum _{i=0}^{m-1} z_i \right) ^{2^{l+1}} \cdot {\text {col}}(z). \end{aligned}$$

Since $\sum _{i=0}^{m-1} z_i \in \mathfrak {m}_n$, we have that $\left( \sum _{i=0}^{m-1} z_i \right) ^{2^l} = 0_R$. Hence $\left( \sum _{i=0}^{m-1} z_i \right) ^{2^{l+1}} = 0_R$, which implies that $\theta _z^{2^{l + 1}} = I_m$.

Now assume that $\theta _z \notin \ker (q^*_{m,R})$. This means that $q^*_{m,R}(\theta _z) \in {\text {CPM}}^*_m(\mathbb {F}_2)$ is not the identity, which means that $q^*_{m,R}(\theta _z)$ has order 2. Hence $\theta _z^2 \in \ker (q^*_{m,R})$, which implies that ${\text {ord}}(\theta _z^2) \mid 2^{l+1}$ as shown earlier. As a result, we have that ${\text {ord}}(\theta _z) \mid 2 \cdot 2^{l+1} = 2^{l+2}$, which concludes the proof. $\square$

## Application: the linear layer of Xoodoo

In this section, we show that the linear layer of Xoodoo can be interpreted as an $R_{4,32}$-linear map, where $R_{4,32}$ is the local circulant ring as in Example 4. Moreover, we introduce DCD-compositions, which are a type of composition with a similar structure as the linear layer of Xoodoo.

### Xoodoo and local circulant modules

The primitive Xoodoo works on a $3 \cdot 128 = 384$ bit state, and its round function is the composition

$$\begin{aligned} \rho _{\text {east}} \circ \chi \circ \iota \circ \rho _{\text {west}} \circ \theta . \end{aligned}$$

The maps $\rho _{\text {west}}, \theta , \rho _{\text {east}}$ are invertible linear maps, $\iota$ is the addition by a constant, and $\chi$ is a non-linear map also used in Keccak-*f*. The composition $\rho _{\text {west}} \circ \theta \circ \rho _{\text {east}}$ is called the **linear layer** of Xoodoo. Details about the specifications of Xoodoo and its component functions can be found in [4].

An important observation is that the linear layer of Xoodoo is in fact an $R_{4,32}$-linear map of the free circulant module $R_{4,32}^3$. To see this, note that the linear maps $\rho _{\text {west}}$, $\theta$ and $\rho _{\text {east}}$ described in [4] can be represented by the matrices

$$\begin{aligned} \theta = \begin{pmatrix} 1 + f &  f &  f \\ f &  1 + f &  f \\ f &  f &  1 + f \end{pmatrix} , \ \ \rho _{\text {west}} = \begin{pmatrix} 1 &  0 &  0 \\ 0 &  X &  0 \\ 0 &  0 &  Z^{11} \end{pmatrix}, \ \ \rho _{\text {east}} = \begin{pmatrix} 1 &  0 &  0 \\ 0 &  Y &  0 \\ 0 &  0 &  X^2 Z^{8} \end{pmatrix}, \end{aligned}$$

all contained in ${\text {M}}_3(R_{4,32})$ where $f = X Z^5 + X Z^{14} \in R_{4,32}$. Thus the linear layer of Xoodoo is represented by the matrix

$$\begin{aligned} \rho _{\text {west}} \circ \theta \circ \rho _{\text {east}}&= \begin{pmatrix} 1 &  0 &  0 \\ 0 &  X &  0 \\ 0 &  0 &  Z^{11} \end{pmatrix} \cdot \begin{pmatrix} 1 + f &  f &  f \\ f &  1 + f &  f \\ f &  f &  1 + f \end{pmatrix} \cdot \begin{pmatrix} 1 &  0 &  0 \\ 0 &  Z &  0 \\ 0 &  0 &  X^2 Z^8 \end{pmatrix} \\&= \begin{pmatrix} 1 + f &  Z \cdot f &  X^2 Z^8 \cdot f \\ X \cdot f &  XZ \cdot (1 + f) &  X^3 Z^8 \cdot f \\ Z^{11} \cdot f &  Z^{12} \cdot f &  X^2 Z^{19} \cdot (1 + f) \end{pmatrix}. \end{aligned}$$

#### Remark 9

We use the letter *Z* as our second variable instead of the usual *Y*, as this corresponds to the lane *z* in the Xoodoo specifications [4]. However in the Sage code, we did use the notation *y*.

#### Proposition 37

The matrices of $\rho _{\text {west}}$, $\theta$ and $\rho _{\text {east}}$ are contained in $\ker \left( q^*_{3,R} \right)$.

#### Proof

Note that $f(1,1) = 1 \cdot 1^5 + 1 \cdot 1^{14} \equiv 2 \equiv 0 \bmod 2$. Using this, we get

$$\begin{aligned} q^*_{3,R}(\theta )&= q^*_{3,R} \begin{pmatrix} 1 + f &  f &  f \\ f &  1 + f &  f \\ f &  f &  1 + f \end{pmatrix} \\&= \begin{pmatrix} 1 + f(1,1) &  f(1,1) &  f(1,1) \\ f(1,1) &  1 + f(1,1) &  f(1,1) \\ f(1,1) &  f(1,1) &  1 + f(1,1) \end{pmatrix} \\&= \begin{pmatrix} 1 &  0 &  0 \\ 0 &  1 &  0 \\ 0 &  0 &  1 \end{pmatrix}, \end{aligned}$$

hence $\theta \in \ker (q^*_{3,R})$. In a similar fashion, we conclude that $\rho _{\text {west}}, \rho _{\text {east}} \in \ker (q^*_{3,R})$. $\square$

By the above proposition together with Corollary 20, we have that

$$\begin{aligned} {\text {ord}}(\rho _{\text {west}} \circ \theta \circ \rho _{\text {east}}) \mid 2^{5+1} = 2^6 = 64, \end{aligned}$$

which is relative low. In fact, we verified using Sagemath that

$$\begin{aligned} {\text {ord}}(\rho _{\text {west}} \circ \theta \circ \rho _{\text {east}}) = 32. \end{aligned}$$

The corresponding code can be found in Appendix A.

### DCD-compositions

We introduce DCD-compositions, which are maps with a similar structure as the linear layer of Xoodoo.

#### Definition 10

Define $D_3^*(R)$ as the set of all invertible diagonal matrices in ${\text {M}}_3(R)$ over a circulant ring *R*, which forms a group under matrix multiplication. We say that a map $\sigma \in {\text {GL}}_3(R)$ is a **DCD-composition** if there exist $\rho _l, \rho _r \in D_3^*(R)$ and $\theta \in {\text {CPM}}^*_3(R)$ such that

$$\begin{aligned} \sigma = \rho _l \circ \theta \circ \rho _r. \end{aligned}$$

#### Remark 10

The linear layer of Xoodoo is a DCD-composition since $\rho _{\text {east}}, \rho _{\text {west}} \in D^*_3(R_{4,32})$ and $\theta \in {\text {CPM}}^*_3(R_{4,32})$.

We present two examples of DCD-compositions. In the first example, we construct a DCD-composition with the same bit-state as the linear layer of Xoodoo (also over $R_{4,32}$), but with the highest possible order of such a DCD-composition.

In the second example, we present a DCD-composition over a non-local circulant ring, which resulted in a higher order.

#### Example 1: DCD-composition over $R_{4,32}$

Let $R = R_{4,32}$, and define the group composition $\mathcal {G}_3(R_{4,32}) = D^*_3(R_{4,32}) \cdot {\text {CPM}}^*_3(R_{4,32})$ which is a subgroup of ${\text {GL}}_3(R_{4,32})$.

##### Theorem 38

For $\sigma \in \mathcal {G}_3(R_{4,32})$, we have that ${\text {ord}}(\sigma ) \mid 2^7$.

##### Proof

Note that $\sigma$ is of the form $\sigma = \prod _{i=1}^n \rho _i \theta _i$ where $\rho _i \in D_3^*(R_{4,32})$ and $\theta _i \in {\text {CPM}}_3^*(R_{4,32})$. Observe that $D_3^*(R_{4,32}) \subset \ker (q^*_{3,R})$, hence $q^*_{3,R}( \sigma ) = q^*_{3,R}( \prod _{i=1}^n \theta _i )$ which is contained in ${\text {CPM}}_3^*(\mathbb {F}_2)$. By Lemma 35, all elements in ${\text {CPM}}_3^*(\mathbb {F}_2)$ either have order 1 or 2. This implies that ${\text {ord}}(\theta )$ must divide $2 \cdot 2^{6} = 2^7$, since the order of all matrices in $\ker (q^*_{3,R})$ divide $2^{1+5} = 2^6$ by Corollary 20 (note that $32 = 2^5$). This concludes the proof. $\square$

Every DCD-composition is contained in $\mathcal {G}_3(R_{4,32})$, which implies that the order cannot exceed $2^7 = 128$.

Consider

$$\begin{aligned} \theta = \begin{pmatrix} 1 + f_1 &  f_1 &  f_1 \\ f_2 &  1 + f_2 &  f_2 \\ f_3 &  f_3 &  1 + f_3 \end{pmatrix} , \ \ \ \ \rho _l = \begin{pmatrix} 1 &  0 &  0 \\ 0 &  X &  0 \\ 0 &  0 &  Z^{11} \end{pmatrix}, \ \ \ \ \rho _r = \begin{pmatrix} 1 &  0 &  0 \\ 0 &  Z &  0 \\ 0 &  0 &  X^2 Z^{8} \end{pmatrix}, \end{aligned}$$

where $f_1 = X Z^5 + X Z^{11} + 1$, $f_2 = X Z^5 + X Z^{11}$ and $f_3 = X Z^5 + X Z^{11} + 1$. We verified using SageMath that ${\text {ord}}(\rho _l \circ \theta \circ \rho _r) = 128$, which is the maximal possible order of such a composition by the above theorem. The corresponding code can be found in Appendix B.

#### Example 2: DCD-composition over $R_n$

Consider circulant rings of the form $R_n = \mathbb {F}_2[X] / \langle X^n - 1 \rangle$, which represents the ring of circulant matrices of dimension *n*.

##### Theorem 39

Let *n* be an odd number, and let $f \in R_n^*$. Then

$$\begin{aligned} {\text {ord}}(f) \mid 2^{{\text {ord}}_n(2)} - 1. \end{aligned}$$

##### Proof

Note that ${\text {ord}}_n(2)$ is well-defined since *n* is odd. Let $f = a_d X^d + a_{d-1} X^{d-1} + \cdots + a_1 X + a_0 \in R_n^*$. Since we work over $\mathbb {F}_2$, we have

14 $$\begin{aligned} f^{2^{{\text {ord}}_n(2)}}&= \big (a_d X^d + a_{d-1} X^{d-1} + \cdots + a_1 X + a_0\big )^{2^{{\text {ord}}_n(2)}} \end{aligned}$$

15 $$\begin{aligned}&= \big (a_d X^d\big )^{2^{{\text {ord}}_n(2)}} + \big (a_{d-1} X^{d-1}\big )^{2^{{\text {ord}}_n(2)}} + \cdots + (a_1 X)^{2^{{\text {ord}}_n(2)}} + a_0^{2^{{\text {ord}}_n(2)}} \end{aligned}$$

16 $$\begin{aligned}&= a_d \left( X^{2^{{\text {ord}}_n(2)}} \right) ^d + a_{d-1} \left( X^{2^{{\text {ord}}_n(2)}} \right) ^{d-1} + \cdots + a_1 X^{2^{{\text {ord}}_n(2)}} + a_0 \end{aligned}$$

By definition, we have $2^{{\text {ord}}_n(2)} \equiv 1 \bmod n$, which implies that

$$\begin{aligned} X^{2^{{\text {ord}}_n(2)}} \equiv X \bmod \langle X^n - 1 \rangle . \end{aligned}$$

Hence we can conclude from Expression (16) that $f^{2^{{\text {ord}}_n(2)}} \equiv f \bmod \langle X^n - 1 \rangle$, implying that $f^{2^{{\text {ord}}_n(2)} - 1} \equiv 1 \bmod \langle X^n - 1 \rangle$ by invertibility of *f*. Thus the order of $f \in R_n^*$ must divide $2^{{\text {ord}}_n(2)} - 1$, which concludes the proof. $\square$

Let us choose $n = 167$. Observe that ${\text {ord}}_{167}(2) = 83$, which by the above theorem means that the highest possible order of elements in $R_{167}^*$ equals $2^{83} - 1$. Consider

$$\begin{aligned} \theta = \begin{pmatrix} 1 + f &  f &  f \\ f &  1 + f &  f \\ f &  f &  1 + f \end{pmatrix} , \ \ \ \ \rho _l = \begin{pmatrix} 1 &  0 &  0 \\ 0 &  X &  0 \\ 0 &  0 &  X^{11} \end{pmatrix}, \ \ \ \ \rho _r = \begin{pmatrix} 1 &  0 &  0 \\ 0 &  X &  0 \\ 0 &  0 &  X^{10} \end{pmatrix}, \end{aligned}$$

where $f = X^6 + X^{15}$. Here we have

$$\begin{aligned} \det (\rho _l \circ \theta \circ \rho _r) = X^{12} \cdot (X^{15} + X^6 + 1) \cdot X^{11} = X^{38} + X^{29} + X^{23}. \end{aligned}$$

By using Sagemath, we verified that $X^{27} + X^{18} + X^{12}$ is invertible (see Appendix C for the code). Since $2^{83} - 1$ is a prime number (it is a Mersenne prime number), it must be the order of $X^{27} + X^{18} + X^{12}$. By Lemma 1, we can conclude that $2^{83} - 1$ divides ${\text {ord}}(\rho _l \circ \theta \circ \rho _r)$.

##### Remark 11

We managed to compute the exact order of the composition $\rho _l \circ \theta \circ \rho _r$, which equals $\left( 2^{83} - 1 \right) \cdot \lambda$ where

$$\begin{aligned} \lambda =&\ 301 \, 541 \, 899 \, 055 \, 510 \, 925 \, 582 \, 216 \, 169 \, 150 \, 861 \, 286 \, 153 \, 081 \, 761 \, 757 \, 331 \, 612 \, 351 \\&867 \, 575 \, 029 \, 327 \, 375 \, 019 \\ \approx&\ 1.33 \cdot 2^{247}. \end{aligned}$$

This is significantly higher than 32. We illustrate a sketch on how we obtained $\lambda$, which requires a bit of mathematical reasoning.

***Computing Strategy (Sketch)***

The ring $R_{167}$ can be naturally embedded in ${\text {GF}}\left( 2^{83} \right) [X] / \langle X^{167} - 1 \rangle$. Note that $X^{167} - 1$ fully splits in ${\text {GF}}\left( 2^{83} \right)$, where we have the decomposition $X^{167} - 1 = \prod _{\zeta \in \mu _{167}} X - \zeta$, where $\mu _{167}$ is the set of 167-th roots of unity. Hence by the Chinese Remainder Theorem, we obtain the isomorphism

$$\begin{aligned} {\text {GF}}\left( 2^{83} \right) [X] / \langle X^{167} - 1 \rangle \rightarrow \bigoplus _{\zeta \in \mu _{167}} {\text {GF}}\left( 2^{83} \right) , \ g \mapsto (g(\zeta ))_{\zeta \in \mu _{167}}. \end{aligned}$$

From this isomorphism, we conclude that

$$\begin{aligned} {\text {GL}}_3 \left( {\text {GF}}\left( 2^{83} \right) [X] / \langle X^{167} - 1 \rangle \right) \cong \bigoplus _{\zeta \in \mu _{167}} {\text {GL}}_3 \left( {\text {GF}}\left( 2^{83} \right) \right) . \end{aligned}$$

By Lagrange, the order of every element in ${\text {GL}}_3 \left( {\text {GF}}\left( 2^{83} \right) [X] / \langle X^{167} - 1 \rangle \right)$ must divide

17 $$\begin{aligned} \#{\text {GL}}_3 \left( {\text {GF}}\left( 2^{83} \right) \right) = \left( 2^{3 \cdot 83} - 1 \right) \cdot \left( 2^{3 \cdot 83} - 2^{83} \right) \cdot \left( 2^{3 \cdot 83} - 2^{2 \cdot 83} \right) , \end{aligned}$$

hence $\lambda$ must be a divisor of (17).

Using Sagemath, we verified that $\lambda \mid \left( 2^{3 \cdot 83} - 1 \right) \cdot \left( 2^{3 \cdot 83} - 2^{83} \right)$. Note that

$$\begin{aligned}&\left( 2^{3 \cdot 83} - 1 \right) \cdot \left( 2^{3 \cdot 83} - 2^{83} \right) \\&= \left( 2^{83} - 1 \right) \cdot \left( 2^{2 \cdot 83} + 2^{83} + 1 \right) \cdot 2^{83} \cdot \left( 2^{2 \cdot 83} - 1 \right) \\&= \left( 2^{83} - 1 \right) \cdot \left( 2^{2 \cdot 83} + 2^{83} + 1 \right) \cdot 2^{83} \cdot (2^{83} + 1) \cdot \left( 2^{83} - 1 \right) \\&= \left( 2^{83} - 1 \right) ^2 \cdot 2^{83} \cdot \left( \left( 2^{2 \cdot 83} + 2^{83} + 1 \right) \cdot \left( 2^{83} + 1 \right) \right) . \end{aligned}$$

Again using Sagemath, we verified that $\lambda \mid \left( 2^{2 \cdot 83} + 2^{83} + 1 \right) \cdot \left( 2^{83} + 1 \right)$. By exhaustive search over the divisors of $\left( 2^{2 \cdot 83} + 2^{83} + 1 \right) \cdot \left( 2^{83} + 1 \right)$, we managed to find $\lambda$. The details of the code used to compute $\lambda$ can be found in Appendix C.

## Discussion: cryptographic implications

The original motivation for this paper was to find a solid mathematical explanation why the order of the linear layer of Xoodoo is relatively low, as this could indicate a potential weakness against invariant subspace attacks [2]. The commutative algebraic reformulation not only provides this explanation, but gave us a deeper insight in the algebraic structure of circulant-like mappings, which is a popular choice for many cryptographic schemes. We believe that we can use our reformulation to find submodules of circulant modules which behave nicely given a module homomorphism. This can mean for example that the submodules stay invariant, or where the images contain a certain structure which remain invariant after multiple propagations. A potentially interesting research topic related to Xoodoo is to find such modules and invariant properties of a given circulant module homomorphism (e.g. the linear layer of Xoodoo), and to which extend they remain invariant when applying the non-linear map $\chi$. This could have interesting cryptographic implications, as we believe such properties can be used to develop potentially effective distinguishers for Xoodoo-like primitives, where its linear layer relies on circulant-like mappings followed by $\chi$.

Another potentially interesting application of this module theoretic approach is to see how this can be applied to study the linear components of the hash function Troika [7], where they work over $\mathbb {F}_3$ instead of the binary field $\mathbb {F}_2$. They adapt a variation of column parity mixers over $\mathbb {F}_3$, which we believe can also be reinterpreted using our module-theoretic setting. This could also be an interesting topic of research related to our work.

## Concluding remarks

There are two main reasons why the order of the linear layer of Xoodoo is relatively low. These being that the linear layer of Xoodoo is contained in $\ker (q^*_{3,R_{4,32}})$, and that the circulant ring $R_{4,32}$ is local. Example 2 of the DCD-compositions demonstrated that for a non-local circulant ring, one can construct DCD-compositions with a much higher order than the linear layer of Xoodoo. An interesting follow up research topic would be to study algebraic properties of non-local circulant rings, and to use these properties to experiment in constructing high order DCD-compositions.
